# Developing Dignity of Risk Education: Exploratory Needs Assessment With Healthcare Staff

**DOI:** 10.1093/geroni/igaf122.2089

**Published:** 2025-12-31

**Authors:** Catherine-Anne Murray, Elizabeth Gillis, Karen Nicholls, Connor Dawe

**Affiliations:** Nova Scotia Health, Halifax, Nova Scotia, Canada; Nova Scotia Health, Halifax, Nova Scotia, Canada; Nova Scotia Health, Halifax, Nova Scotia, Canada; Nova Scotia Health Implementation Science, Halifax, Nova Scotia, Canada

## Abstract

The Dignity of Risk (DoR) concept is being applied more to the care of older adults living with frailty, as healthcare systems are being challenged to broaden perspectives and policy to include a human rights lens to care provision. This initiative aims to develop and provide education for health care staff, based on a needs assessment and DoR evidence from the literature. To explore staff perspectives on DoR in the care of older adults, more than 600 surveys were completed at acute care and community sites across Nova Scotia. Main findings include: Most staff have little to no previous knowledge of the DoR, however most felt it moderately-very important to implement into care. Most staff identified the following barriers to implement DoR into practice: a hyperfocus on safety culture, paternalistic attitudes, fear of liability, and ageism. Other challenges included cognitive issues of the older adult and lack of support from manager and team members. Staff identified DoR facilitators as: policies and guidelines reinforcing same, skills in risk management conversations with caregivers, and understanding how decision-making abilities of the older adult can affect DoR application. Education via participatory workshops was developed and provided. Topics included defining DoR and its value in older adult care, ageism, overprotective attitudes, liability, using an informed strength-based risk management approach in care planning, and building confidence in difficult conversations with caregivers. Post workshop surveys were completed and main findings shared in following abstract.

